# Changes in hemodynamics associated with metabolic syndrome are more pronounced in women than in men

**DOI:** 10.1038/s41598-019-54926-0

**Published:** 2019-12-05

**Authors:** Pauliina Kangas, Antti Tikkakoski, Jarkko Kettunen, Arttu Eräranta, Heini Huhtala, Mika Kähönen, Kalle Sipilä, Jukka Mustonen, Ilkka Pörsti

**Affiliations:** 1 Faculty of Medicine and Health Technology, FI-33014 Tampere University, Tampere, Finland; 2 Department of Clinical Physiology and Nuclear Medicine, Tampere University Hospital, P.O. Box 2000, FI-33521 Tampere, Finland; 3 Faculty of Social Sciences, FI-33014 Tampere University, Tampere, Finland; 4 Department of Internal Medicine, Tampere University Hospital, P.O. Box 2000, FI-33521 Tampere, Finland

**Keywords:** Cardiovascular biology, Hypertension

## Abstract

The increase in cardiovascular risk associated with metabolic syndrome (MS) seems higher in women than in men. We examined hemodynamics during head-up tilt in 252 men and 250 women without atherosclerosis, diabetes, or antihypertensive medication, mean age 48 years, using whole-body impedance cardiography and radial pulse wave analysis. MS was defined according to Alberti *et al*. 2009. Men and women with MS presented with corresponding elevations of systolic and diastolic blood pressure (10-14%, p ≤ 0.001) versus controls. Supine pulse wave velocity (16–17%, p < 0.001) and systemic vascular resistance (7–9%, p ≤ 0.026), and upright cardiac output (6–11%, p ≤ 0.008) were higher in both MS groups than controls. Elevation of supine aortic characteristic impedance was higher in women than in men with MS (16% vs. 8%, p = 0.026), and in contrast to men, no upright impedance reduction was observed in women. When upright, women but not men with MS showed faster return of reflected pressure wave (p = 0.036), and smaller decrease in left cardiac work (p = 0.035) versus controls. The faster upright return of reflected pressure, lower upright decrease in left cardiac work, and higher elevation of aortic characteristic impedance may contribute to the greater increase in MS-related cardiovascular risk in women than in men.

## Introduction

Metabolic syndrome (MS) is defined as a cluster of abnormalities in glucose tolerance, lipid profile, blood pressure (BP), and amount of visceral adipose tissue^1^. When compared with subjects without MS, a 5-fold increase in the risk of type 2 diabetes mellitus, and a 2-fold risk of developing cardiovascular (CV) disease over the next 5–10 years, is observed in subjects with MS^1^. Due to the increasing incidence of overweight, the prevalence of MS has strongly increased over the last decades^2^.

The mechanisms underlying the elevated CV risk in MS have been under active investigation, and several studies have addressed the associated hemodynamic alterations. Increased large arterial stiffness^3^, aortic pulse pressure^4^, and systemic vascular resistance (SVR) have been associated with MS^5^. Also decreased left ventricular stroke index, and impaired left ventricular systolic and diastolic functions have been found in individuals with MS^5,6^. Obesity and MS are also associated with arrhythmias^7–9^, and increased arrhythmic burden is not only observed in MS patients with a failing heart but even in subjects with normal cardiac morphology^10,11^. When compared with subjects without MS, the prevalence of left ventricular hypertrophy^12^, and even the risk of CV mortality^13^, are more pronounced in women than in men with MS. Furthermore, the predisposing effect of MS on early atherosclerosis is higher in women than in men^14^. The underlying mechanisms are not completely understood.

Recently, we found that a clear difference in cardiovascular responses between sexes was higher workload for the heart in men in the upright position, a finding that was not explained by known cardiovascular risk factors or hormonal differences^15^. To our knowledge, the regulation of upright hemodynamics has not been examined in subjects with MS. In the present study, our objective was to examine whether there are differences in the MS-associated changes in cardiovascular function between men and women. To test this hypothesis, non-invasive hemodynamics were recorded in supine position and during passive head-up tilt in men and women with MS and respective controls groups.

## Methods

### Study subjects

This study is part of an ongoing clinical study on hemodynamics in the University of Tampere (DYNAMIC-study, clinical trial registration NCT01742702). The participants gave written informed consent, and the study was approved by the Ethics Committee of Tampere University Hospital (study code R06086M) conforming to the principles outlined in the Declaration of Helsinki. The participants were enrolled from adult patients (age ≥18 years) treated at Tampere University Hospital, and enrollment was also made via announcements in offices of local occupational health care providers, Varala Sports Institute, among employees of the Tampere University and Tampere University Hospital, while 2 announcements were published in local newspapers. Those who agreed to participate were recruited in the order in which their contact information was available to the research nurses. The present population was screened from 956 volunteers. The exclusion criteria were diagnosed diabetes, atherosclerosis, cardiac insufficiency, cerebrovascular disease, heart rhythm other than sinus; any acute health problem; and use of antihypertensive drugs or other medications with influences on hemodynamics (α_1_-adrenoceptor blockers for prostate problems, β-blocker eye drops for glaucoma, β_2_-adrenoceptor agonists, and digoxin). The study group consisted of 502 subjects aged 24–72 years (mean 48, SD 9.3). Lifestyle habits, family history, medical history, and use of medicines were recorded, and clinical cardiovascular status was examined. Laboratory tests were taken 11 ± 2 days (mean ± 95% confidence interval (CI) of the mean) before the hemodynamic recordings in order to exclude concurrent illnesses that would interfere with the interpretation of the results.

MS was defined according to Alberti *et al*.^1^, so that ≥3 of the following criteria were met: waist circumference ≥94 cm (men) and ≥80 cm (women); triglycerides ≥1.7 mmol/l; high density lipoprotein (HDL) cholesterol <1.0 mmol/l (men) and <1.3 mmol/l (women); systolic BP ≥130 mmHg and/or diastolic BP ≥85 mmHg; fasting plasma glucose ≥5.6 mmol/l^1^. The subjects were allocated to 4 groups: men without MS (Men-control, n = 133), men with MS (Men-MS, n = 119), women without MS (Women-control, n = 196), and women with MS (Women-MS, n = 54).

Altogether 181 subjects (36% of the study population) used medications (Table 1). Thirteen were on statins for dyslipidemia, 75 female subjects (30%) used systemic estrogen, progestin, or their combination (contraception or hormone replacement therapy), and one subject used tibolone. There was no difference in the use of female hormones between the Women-control and Women-MS groups (p = 0.990). One subject without symptoms used warfarin for anti-phospholipid syndrome. Also other medications (acetylsalicylic acid, selective serotonin re-uptake inhibitors, antihistamines, thyroid hormones, proton pump inhibitors, and intranasal or inhaled corticosteroids) were used by individual subjects (see Table 1). Information about alcohol intake was missing from 13 subjects.

**Table 1:** Medications used regularly by the study participants (number of participants and percentages with each type of medication).

	Men-control n = 133	Men-MS n = 119	Women-control n = 196	Women-MS n = 54
Acetylsalicylic acid	2 (1.5%)	2 (1.7%)	1 (0.5%)	2 (3.7%)
Acyclovir	1 (0.8%)	0	0	0
Alendronate	0	0	1 (0.5%)	0
Allopurinol	0	1 (0.8%)	0	1 (1.9%)
Amitriptyline	0	0	2 (1.0%)	0
Amoxicillin	0	0	0	1 (1.9%)
Antidepressant (SSRI or SNRI)	3 (2.3%)	5 (4.2%)	11 (5.6%)	6 (11.1%)
Antihistamine	0	2 (1.7%)	8 (4.1%)	1 (1.9%)
Benzodiazepine	0	0	2 (1.0%)	0
Carbamazepine	0	0	0	1 (1.9%)
Carbimazole	0	0	1 (0.5%)	0
Cholestyramine	0	1 (0.8%)	0	0
Dehydroepiandrosterone	1 (0.8%)	0	0	0
Doxycycline (low dose)	0	1 (0.8%)	0	0
Ezetimibe	0	1 (0.8%)	0	0
Female hormones				
Systemic (including tibolone and levonorgestrel via intrauterine device)	0	0	59 (30.1%)	16 (29.6%)
Topical			4 (2.0%)	1 (1.9%)
Glucosamine	2 (1.5%)	1 (0.8%)	2 (1.0%)	1 (1.9%)
Hydroxocobalamin	1 (0.8%)	0	0	0
Hydroxycarbamide	1 (0.8%)	0	0	0
Intranasal or inhaled corticosteroid	1 (0.8%)	3 (2.5%)	9 (4.6%)	1 (1.9%)
Isotretinoin	0	0	1 (0.5%)	0
Letrozole	0	0	1 (0.5%)	0
Levetiracetam	0	0	1 (0.5%)	0
Liothyronine	0	0	0	1 (1.9%)
Mefloquine	0	0	1 (0.5%)	0
Melatonin	1 (0.8%)	0	0	1 (1.9%)
Mepacrine	0	1 (0.8%)	0	0
Mesalazine	0	0	0	1 (1.9%)
Methenamine hippurate	0	0	1 (0.5%)	0
Montelukast	0	0	1 (0.5%)	0
Non-steroidal anti-inflammatory drug	1 (0.8%)	1 (0.8%)	3 (1.5%)	1 (1.9%)
Oxcarbazepine	0	0	1 (0.5%)	0
Pramipexole	0	0	1 (0.5%)	0
Prednisolone	1 (0.8%)	0	0	0
Pregabalin	1 (0.8%)	0	0	1 (1.9%)
Proton pump inhibitor	5 (3.8%)	5 (4.2%)	0	3 (5.6%)
Quetiapine	0	0	1 (0.5%)	0
Statin	5 (3.8%)	5 (4.2%)	0	3 (5.6%)
Tafluprost	0	0	0	1 (1.9%)
Tamoxifen	0	0	1 (0.5%)	0
Thyroxine	1 (0.8%)	0	13 (6.6%)	2 (3.7%)
Valproate	0	0	1 (0.5%)	0
Varenicline	0	0	1 (0.5%)	1 (1.9%)
Vitamin D supplementation	12 (9.0%)	5 (4.2%)	18 (9.2%)	8 (14.8%)
Warfarin	0	1 (0.8%)	0	0

SNRI indicates serotonin-norepinephrine reuptake inhibitor; SSRI, selective serotonin reuptake inhibitor.

### Hemodynamic measurements

The subjects were advised to refrain from caffeine containing products, smoking and heavy meals for ≥4 hours, and from alcohol for ≥24 hours prior to the recordings^15,16^. The recordings took place between 08:30 a.m. and 04:00 p.m. on working days. A brief introductory passive head-up tilt on a tilt-table was performed with ≥5 minutes of rest in the supine position before and after the head-up tilt. Then hemodynamics were recorded by a trained research nurse in a temperature-controlled laboratory during two consecutive 5-minute periods with continuous capture of data: 5 minutes supine on a tilt table, followed by passive head-up tilt to ≥60 degrees for 5 minutes^15–17^. For the definition of MS, the average systolic and diastolic BPs of the last supine 3 minutes were used. The detailed description of the protocol has been published^15–17^, and the repeatability and reproducibility of the measurements has been demonstrated (repeatability index in two consecutive measurements for augmentation index (AIx) 95% supine and 95% upright, for stroke volume 99% supine and 99% upright; reproducibility index on four separate days for AIx 78% supine and 70% upright, for stroke volume 89% supine and 93% upright)^17^.

### Pulse wave analysis, PWA

BP and pulse wave form were continuously captured from the radial pulsation by a tonometric sensor (Colin BP-508T, Colin Medical Instruments Corp., USA). Radial BP signal was calibrated approximately every 2.5 minutes by contralateral brachial BP measurements. Variables of central pressures and wave reflection (forward wave amplitude, subendocardial viability ratio (SEVR, ratio of diastolic area/min to systolic area/min); aortic BP, reflection time, AIx, and AIx related to heart rate 75/min (AIx@75)) were derived online using a pulse wave monitoring system (SphygmoCor PWMx, AtCor medical, Australia), and a previously validated generalized transfer function^18^. The left arm with the tonometric sensor was abducted to 90 degrees in a support, which held the arm and the wrist steady at the level of the heart in both supine and upright positions^15,16^.

### Whole-body impedance cardiography

Whole-body impedance cardiography (CircMon^R^, JR Medical Ltd., Tallinn, Estonia), was used to determine beat-to-beat heart rate, cardiac output and aortic-to-popliteal pulse wave velocity (PWV)^19,20^. SVR and left cardiac work (LCW) were calculated from the tonometric BP and cardiac output measured by the CircMon^R^. Average supine central venous pressure is ~3–4 mmHg, while during upright position the value is close to zero mmHg^21–23^. As central venous pressure was not measured in the present study, the formula for SVR estimation did not include this variable and was calculated as follows: SVR = 79.96 * mean arterial BP/cardiac output; 79.96 was the conversion factor from mmHg/L/min to dyn*s/cm^5^. LCW was calculated as 0.0143 × (mean arterial BP − pulmonary artery occlusion pressure) × cardiac output. Pulmonary artery occlusion pressure was assumed to be 6 mmHg (normal), and 0.0143 was the conversion factor of pressure from millimeter of mercury to centimeters of water, volume to density of blood, centimeters to meters, and conversion from gram to gram force^16^. Time-domain estimate of aortic characteristic impedance [(pressure at inflection point - diastolic aortic BP) * systolic time)/(stroke volume * 2)] was calculated according to Chemla *et al*. so that aortic BP and systolic time were derived from pulse wave analysis and stroke volume from impedance cardiography^24^.

When using the CircMon^R^ whole body impedance cardiography, the cardiac output values of correlate well with the thermodilution method (bias 0.00 l/min, 95% CI −0.26 to 0.26) and the direct oxygen Fick method (bias −0.32 l/min, 95% CI −0.69 to 0.05)^25^, corresponding upright reductions in cardiac output are observed when compared with thermodilution^21^, the upright stroke volume shows good correlation with 3-dimensional echocardiography (r = 0.781, bias 4.1 ml, 95% CI −2.2 to 10.4)^16^, and the PWV values show good correlation with the tonometric method (r = 0.82, bias 0.02 m/s, 95% CI −0.21 to 0.25)^20^. Due to technical problems, PWV from one subject, aortic characteristic impedance and AIx@75 from 3 subjects supine and 3 subjects upright, time to the return of the reflected wave from 3 subjects supine and 4 subjects upright, were missing.

### Laboratory tests

Fasting plasma glucose, triglycerides, total cholesterol, HDL and low-density lipoprotein (LDL) cholesterol, and creatinine were measured using Cobas Integra 700/800 or Cobas 6000 (Roche Diagnostics, Basel, Switzerland), and insulin using electrochemiluminescence immunoassay (Cobas e 411, Roche Diagnostics). Quantitative insulin sensitivity check index (QUICKI) was calculated^26^. Estimated glomerulus filtration rate (eGFR) was determined using the CKD-EPI formula^27^. A standard 12-lead electrocardiogram (ECG) was recorded and Cornell voltage QRS duration product calculated^28^. LDL cholesterol values from six subjects, and ECG from one subject, were missing.

### Statistical analyses

The characteristics between the control and MS groups in each sex (Table 2), and the hemodynamic profiles of the MS groups, were compared using independent samples t-test. The skewed triglyceride distribution was logarithmically transformed before the analyses. Alcohol intake, smoking habits, and use of female hormones were compared using Mann-Whitney U-test and Pearson Chi-Square test.

**Table 2:** Clinical and metabolic characteristics in the study groups.

Variable	Men-control	Men-MS	Women-control	Women-MS
Number of subjects	133	119	196	54
Age (years)	48 ± 10	49 ± 9	47 ± 9	50 ± 10*
BMI (kg/m^2^)	26 ± 3	30 ± 3*	25 ± 4	30 ± 5*
Weight (kg)	86 ± 12	95 ± 11*	70 ± 13	81 ± 14*
Height (cm)	181 ± 6	179 ± 5*	166 ± 6	165 ± 5
Waist circumference (cm)	95 ± 9	105 ± 8*	85 ± 12	96 ± 12*
Systolic blood pressure (mmHg)	129 ± 15	144 ± 16*	124 ± 18	141 ± 18*
Diastolic blood pressure (mmHg)	74 ± 10	84 ± 10*	72 ± 12	80 ± 12*
Pulse wave velocity (m/s)	8.50 ± 1.8^#^	9.88 ± 2.0*	7.87 ± 1.4	9.21 ± 1.9*
Stroke volume supine (ml)	99 ± 15	97 ± 11	81 ± 15	77 ± 14
Stroke volume upright (ml)	76 ± 10	78 ± 9	56 ± 8	59 ± 9
**Smoking status**				
Never smoked (n/%)	70/52%	57/48%	123/63%	29/54%
Current smoker (n/%)	21/16%	14/12%	24/12%	6/11%
Previous smoker (n/%)	42/32%	48/40%	49/25%	19/35%
Alcohol intake (standard doses/week)	4 (1–9)	4 (1–10)	2 (1–3)	2 (1–4)
Creatinine (µmol/l)	83 ± 12	81 ± 11	66 ± 9	62 ± 9*
eGFR (ml/min/1.73 m^2^)	95 ± 13	95 ± 12	95 ± 13	98 ± 13
Fasting plasma glucose (mmol/l)	5.4 ± 0.4	5.9 ± 0.4*	5.2 ± 0.4	5.8 ± 0.5*
Total cholesterol (mmol/l)	5.1 ± 0.9	5.7 ± 1.1*	5.1 ± 1.0	5.7 ± 0.8*
Triglycerides (mmol/l)	1.0 (0.7–1.4)	1.8 (1.1–2.4)*	0.9 (0.6–1.1)	1.5 (1.1–2.1)*
High-density lipoprotein cholesterol (mmol/l)	1.5 ± 0.3	1.2 ± 0.3*	1.9 ± 0.4	1.5 ± 0.4*
Low-density lipoprotein cholesterol (mmol/l)	3.1 ± 0.9	3.7 ± 0.9*	2.8 ± 0.9	3.5 ± 0.7*
Quantitative insulin sensitivity check index	0.365 ± 0.045	0.342 ± 0.040*	0.373 ± 0.041	0.338 ± 0.031*
Cornell voltage product in ECG (ms*mm)	1621 ± 817	1772 ± 557	1546 ± 516	1764 ± 518*

Values are means ± SD except the values for smoking, which are the number of cases and percentages, and the values for triglycerides and alcohol intake, which are shown as medians (lower and upper quartiles) due to skewed distribution. Men-control, men without MS; Men-MS, men with MS; Women-control, women without MS; Women-MS, women with MS; *p < 0.05 MS vs control group; BMI, body mass index; eGFR, estimated glomerulus filtration rate^27^; ^#^n = 132 for pulse wave velocity in the Men-control group.

Mean values of the hemodynamic variables during each minute of recording in individual study subjects were calculated, and the generalized estimating equation (GEE) adjusted for age was applied. This method enabled the analyses of repeated measurements over the 10 min recording period to examine the influences of MS, sex, and their interaction with posture on the hemodynamic variable of interest. Linear scale response was applied, and the autoregressive option was chosen for the correlation matrix, as successive serial measures of hemodynamics in individual participants are auto-correlated.

In order to compare the hemodynamic profiles in men and women with MS, the average values of the last 3 minutes of the supine and upright periods were used due to the representative and stable signal during this period^29^ (please see also figures). In each participant with MS, the mean value of the last 3 minutes of supine or upright recording for each variable was calculated as percentage of the respective mean value in the whole corresponding control group (control men or control women). Then the percentage values between men and women with MS were compared.

The results in Table 2 are reported as means and standard deviations (normally distributed variables), medians and lower and upper quartiles (variables with skewed distribution), or numbers of cases and percentages (categorical variables). The figures are depicted as means and 95% CI for the mean. All testing was two-sided, and p-values < 0.05 were considered significant. All data were analyzed using IBM SPSS Statistics, software version 25 (Armonk, New York, USA).

## Results

### Study population

Mean age did not differ between the Men-control and the Men-MS groups (p = 0.254), but was 3 years higher in the Women-MS than in the Women-control group (p = 0.035) (Table 2). BMI, waist circumference, and BP were higher in both MS groups than in the control groups (p < 0.001 for all). Aortic-to-femoral PWV was about 16–17% higher in men and women with MS than in the respective control groups without MS (p < 0.001 for all) (Table 2). Smoking and alcohol use did not differ between the MS and the control groups (p > 0.1 for all).

The eGFR value did not differ between the MS and the respective control groups (p > 0.21 for men and women). As expected, fasting plasma glucose, total and LDL cholesterol, and triglycerides were higher, while HDL cholesterol and QUICKI were lower, in subjects with MS than in subjects without MS (p < 0.001 for all). In women with MS, Cornell voltage product was higher than in women without MS (p = 0.007), while in men the difference between the groups was not significant (p = 0.092).

### Hemodynamics in MS versus control groups

**Figure 1:**
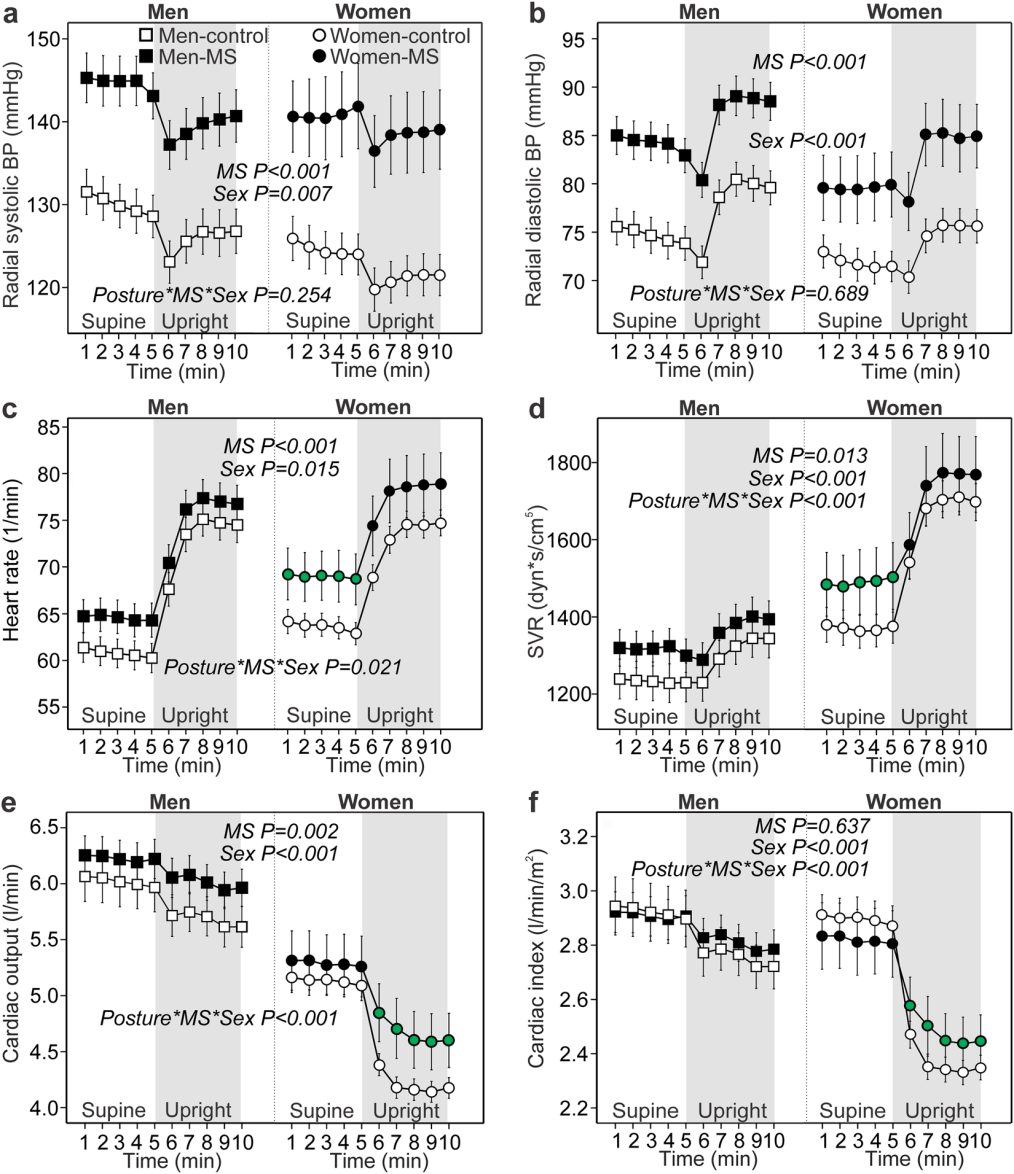
Blood pressure (BP) and variables defining BP level: radial systolic BP (**a**), radial diastolic BP (**b**), heart rate (**c**), systemic vascular resistance (SVR) (**d**), cardiac output (**e**), and cardiac index (**f**); means and 95% confidence intervals of the mean for each minute of recording; age-adjusted p-values calculated using general estimating equations; significant interactions between posture, metabolic syndrome (MS), and sex (Posture_*_MS_*_Sex) shown by green symbol color; n = 133 in men without metabolic syndrome (MS), n = 119 in men with MS; n = 196 in women without MS, n = 54 in women with MS.

**Figure 2:**
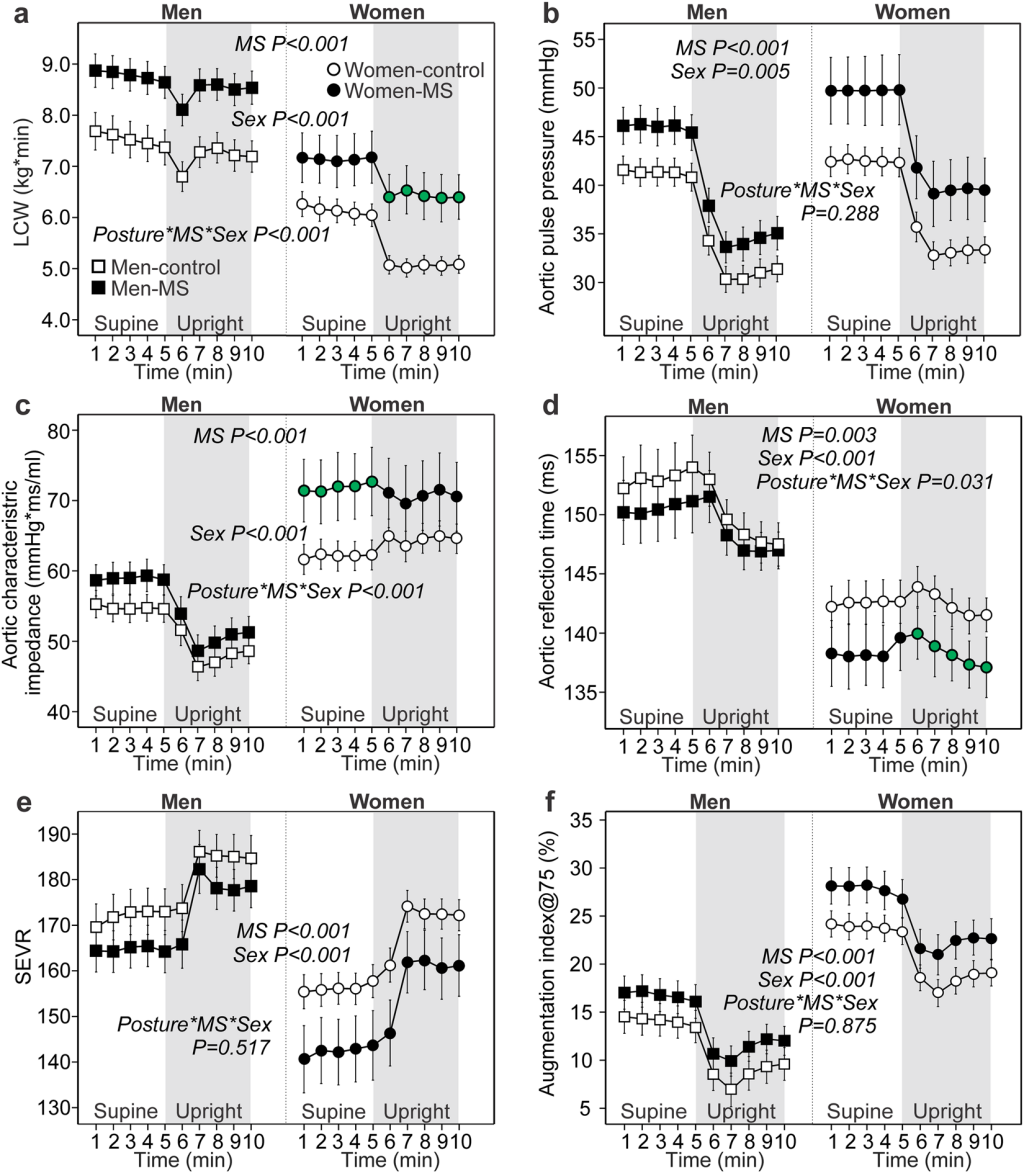
Variables related to cardiac workload and wave reflection: left cardiac work (LCW) (**a**), aortic pulse pressure (**b**), aortic characteristic impedance (**c**), time to return of the reflected wave (**d**), subendocardial viability ratio (SEVR) (**e**), and augmentation index related to heart rate 75/min (**f**); means and 95% confidence intervals of the mean for each minute of recording; age-adjusted p-values calculated using general estimating equations; significant interactions between posture, metabolic syndrome (MS), and sex (Posture_*_MS_*_Sex) shown by green symbol color; n = 132–133 in men without metabolic syndrome (MS), n = 118–119 in men with MS; n = 196 in women without MS, n = 51–54 in women with MS.

During the 10-minute recording protocol (5 minutes supine, 5 minutes of head-up tilt) radial systolic and diastolic BP (Fig. 1a,b), heart rate (Fig. 1c), SVR (Fig. 1d), cardiac output (Fig. 1e), LCW (Fig. 2a), aortic pulse pressure (Fig. 2b), aortic characteristic impedance (Fig. 2c), and AIx@75 (Fig. 2f) were higher in men and women with MS than in the respective control groups. However, cardiac output values related to body surface area (cardiac index) did not differ between the MS groups and the respective control groups (Fig. 1f). The time to the return of the reflected wave was shorter (Fig. 2d) and SEVR was lower (Fig. 2e) in both MS groups than in the respective control groups.

### Hemodynamics and sex

All of the hemodynamic variables were different between sexes. Radial systolic and diastolic BP (Fig. 1a,b), cardiac output (Fig. 1e), cardiac index (Fig. 1f), LCW (Fig. 2a), and SEVR (Fig. 2e) were lower, while aortic reflection time was shorter (Fig. 2d) in women than in men. In contrast, heart rate (Fig. 1c), SVR (Fig. 1d), aortic pulse pressure (Fig. 2b), aortic characteristic impedance (Fig. 2c), and AIx@75 (Fig. 2f) were higher in women than in men.

### Hemodynamics and posture, interactions between posture and sex

With the exception of aortic characteristic impedance in women, all of the hemodynamic variables changed significantly in response to head-up tilting from supine to upright posture (Figs. 1, 2, p < 0.001 for changes in all variables, p-values not shown in figures). Supine and upright stroke volumes are presented in Table 2.

A significant interaction between sex and posture was observed in some variables. In response to the change from supine to upright position, women presented with higher increase in SVR (Fig. 1d); more pronounced decreases in cardiac output, cardiac index and LCW (Figs. 1e,f, 2a); and no decrease in aortic characteristic impedance (Fig. 2c) when compared with men (p < 0.001 for all, p-values not shown in figures). In men the evaluated aortic characteristic impedance was significantly reduced in the upright position when compared with the supine values (p < 0.001).

### Interactions between metabolic syndrome, posture and sex

A significant interaction between the MS, posture and sex was observed in the following variables: women with MS presented with increased supine heart rate, SVR, and aortic characteristic impedance (Figs. 1c,d, 2c); increased upright cardiac output, cardiac index and LCW (Figs. 1e,f, 2a), and shortened upright aortic reflection time (Fig. 2d). Of note, in men none of the changes of the hemodynamic variables in response to upright posture differed between the Men-MS and Men-control groups.

### Profiles of the MS-related hemodynamic changes in men and women

In both sexes MS was characterized by 10-14% higher supine and upright systolic and diastolic BP (Table 2, Fig. 1) than in the respective control groups.

**Figure 3:**
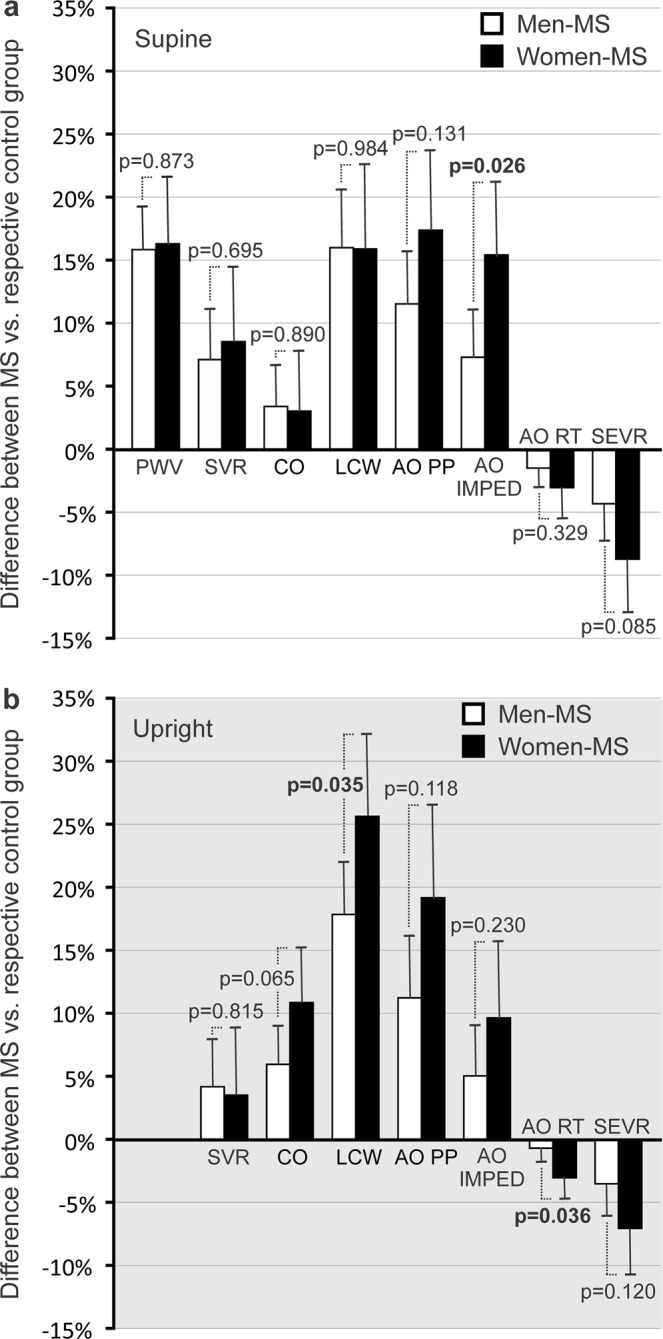
Bar graphs show percent differences in pulse wave velocity (PWV), systemic vascular resistance (SVR), cardiac output (CO), left cardiac work (LCW), aortic pulse pressure (AO PP), aortic characteristic impedance (AO IMPED), aortic reflection time (AO RT), and subendocardial viability ratio (SEVR) in the MS groups versus respective controls, p-values adjusted for age. In each participant with MS, the mean value of the last 3 minutes of supine or upright period of each variable was calculated as percentage of the respective mean value in the whole corresponding control group of participants.

Although MS was associated with a similar rise in PWV (a variable that was only recorded in the supine position) in women and men (16.6 ± 5.5% vs. 16.1 ± 3.5%, respectively, p = 0.873, Fig. 3), supine aortic characteristic impedance was more increased in women than in men with MS (16.0 ± 6.0% vs. 7.5 ± 3.9%, respectively, Fig. 3, p = 0.026). The supine percent changes in the other hemodynamic variables were not significantly different between the Men-MS and Women-MS groups (Fig. 3).

The upright increase in LCW (18% vs. 26%, p = 0.035) and decrease in aortic reflection time (0.6% vs. 3.0%, p = 0.036) were more pronounced in women than in men with MS (Fig. 3). The percent changes in the other hemodynamic variables in the upright position were not significantly different between men and women with MS.

## Discussion

In this study we evaluated hemodynamic changes associated with MS in 502 subjects. We found corresponding increases in PWV, and supine and upright BP in men and women with MS. Still, higher increase in the evaluated supine aortic characteristic impedance was observed in women with MS. During the head-up tilt, women with MS presented with shortened time to the return of the reflected wave and higher increase in LCW than men with MS. The GEE-analyses also uncovered significant interactions between MS and female sex in the upright cardiac output and LCW, indicating more pronounced changes in these variables than in men with MS. Altogether, comparable MS-related increases in BP and large arterial stiffness were associated with hemodynamic changes that potentially burden the heart more in women. Of note, in contrast to men, aortic characteristic impedance was not decreased in the upright position in women. The present results suggest that increases in both SVR and cardiac output contribute to the elevation of BP in MS.

The MS-associated increase in CV risk appears to be higher in women than in men^12,13^, while MS adversely influences cardiovascular morbidity in subjects with primary hypertension independent of its individual components^30^. The pathophysiology of the hemodynamic changes in MS is not completely clear, but an important factor is increased large artery stiffness^3,31^. In the present study PWV was 16–17% higher in men and women with MS than in the control subjects.

In the proximal aorta characteristic impedance regulates the relationship between pressure and flow^24,32^. These variables are also influenced by aortic reservoir characteristics^33–36^, although the matter remains controversial^37,38^. Stiffening of the aorta increases the impedance to flow, while aortic impedance is more sensitive to changes in vessel radius than PWV^24^. In the present study, the increase in supine aortic characteristic impedance was higher in women than in men with MS (16.0% vs. 7.5%, respectively). As the diameter and length of the large arteries are lesser in women than in men^39^, these findings may be attributed to the influences of arterial stiffening upon the smaller aortic size of women. Previously, aortic characteristic impedance and AIx were higher, and time to the return of the reflected wave was shorter, in elderly 272 women than in 189 men^40^.

AIx is higher in women than in men due to shorter stature and smaller large artery size^41–44^. The AIx as a parameter of wave reflection is influenced by ejection time, heart rate, arterial diameter, wall elasticity, wall thickness, arterial branching, and resistance to flow in small arteries^39^. In the present study, AIx was higher, and the time to the return of the reflected pressure wave was shorter, in women than in men both supine and upright. In contrast to men, aortic characteristic impedance was not reduced in the upright position in women. Previous results indicate that SVR may not directly affect wave reflection but rather via changes in BP that have a secondary influence on stiffness, and that blood vessel geometry has a more important role in wave reflection than SVR^45^. However, higher supine SVR and more pronounced upright increase in SVR may contribute to higher wave reflections in women.

MS is associated with increased left ventricular mass and impaired systolic and diastolic function^6,46^. Women with MS may be more susceptible to these changes than men^12,47^, possibly due to the higher aortic characteristic impedance^40^. In 2945 subjects, increased aortic characteristic impedance was associated with worse left ventricular global longitudinal strain, however in adjusted analyses this relation was only observed in women^48^. In the present study, supine LCW was increased by 16% in both sexes with MS, but in the upright position the increase in LCW was higher in women than in men (27% vs. 18%, respectively). In addition, supine (−4% vs. −9%, p = 0.085) and upright (−4% vs. −7%, p = 0.120) decreases in SEVR, a variable evaluating myocardial oxygen supply versus demand^49^, were numerically higher in women than in men with MS. When compared with respective control groups, Cornell voltage product was also higher in women but not in men with MS. Previously, lower SEVR was attributed to lower diastolic pressure-time integral and shorter diastole in female than male subjects aged 2–81 years^44^. Not surprisingly, increased arrhythmic burden has been reported in patients with MS^10,11^. Serum analysis of biomarkers like B-type natriuretic peptide and troponin-I can be used to predict clinical outcomes in patients with MS who suffer from cardiac failure^50^.

Sympathetic overdrive has been linked with MS, and autonomic imbalance may contribute to the increased CO, SVR and BP in subjects with MS^51,52^. The changes in autonomic tone related to MS may also be more pronounced in women than in men^52–55^. Of note, the alterations in autonomic tone in subjects who are overweight, a characteristic feature of MS, show remarkable disparity^56^. Overweight subjects may have normal cardiac sympathetic activity and neuronal noradrenaline uptake, while afferent renal sympathetic activity may still be increased^56^. Decreased parasympathetic activity is also a putative cause for an imbalance in autonomic function^57,58^. We recently found that reduced total and high frequency power of heart rate variability in the upright position may partially explain why the relative increase in cardiovascular risk associated with MS is greater in women than in men^53^. Further studies on the sex-related differences of autonomic tone in MS are warranted.

Different levels of sex hormones and putative changes in the sex hormone profiles are prime candidates for the hemodynamic differences between men and women with MS. In men, MS is associated with reduced testosterone levels^59^, while in women the situation is reversed and testosterone levels are increased in subjects with MS^60^. The sex-related differences in testosterone metabolism potentially influence the hemodynamic responses in men and women with MS, and make an interesting subject for future investigations.

Our study has limitations. (1) The observational design does not allow conclusions about causal relationship. (2) The age differences among the study population comprising 252 men and 250 women were rather large. (3) The non-invasive measurements required mathematical processing and simplification of physiology^25^. Pulmonary artery occlusion pressure was not measured and was assumed to be normal. Supine central venous pressure is normally about 3–4 mmHg, while the upright value is close to zero mmHg^21–23^. As this variable was not measured either, central venous pressure was not included in the formula to calculate SVR. (4) The formula for the estimation of aortic impedance may be more suitable for invasive measurements than tonometric recordings^24^. (5) Although subjects using medications with direct influences on hemodynamics were excluded, the other medications used by 36% of the study population may have influenced the results. Importantly, the use of female hormones did not differ between women with and without MS (Table 1). (6) Information about the phase of the menstrual cycle in the female subjects was not available. (7) The criteria of Alberti *et al*. were applied for the definition of MS^1^, instead of the definition by National Cholesterol Education Program^61^. With the Alberti *et al*. criteria, healthier subjects are defined to have MS. Despite this, the results showed clear hemodynamic changes associated with MS.

In summary, men and women with MS had higher BP than the control subjects without MS. This was probably explained by higher SVR, higher cardiac output, and higher arterial stiffness in subjects with MS. Several of the MS-related changes in hemodynamics seemed more pronounced in women than in men. When compared with the MS-related findings in men, women with MS presented with smaller decreases in cardiac output and LCW in the upright position than women without MS, and shortened time to the return of the reflected pressure wave. Women with MS had also a more pronounced increase in aortic characteristic impedance for a similar increase in BP and arterial stiffness than men with MS. These changes that influence the workload to the heart may contribute to the higher increase in CV risk associated with MS in women.

## Data Availability

Analyses and generated datasets during the current study are not available publicly as our clinical database contains several indirect identifiers and the informed consent obtained does not allow publication of individual patient data. The datasets are available from the corresponding author on reasonable request.
